# Combined application of hot water treatment and eucalyptus leaf extract postpones seneṣcence in harvested green chilies by conserving their antioxidants: a sustainable approach

**DOI:** 10.1186/s12870-023-04588-y

**Published:** 2023-11-18

**Authors:** Muhammad Wasim Haider, Muhammad Nafees, Rashid Iqbal, Sajid Ali, Habat Ullah Asad, Farrukh Azeem, Muhammad Arslan, Muhammad Habib Ur Rahman, Abdel-Rhman Z. Gaafar, Mohamed S. Elshikh

**Affiliations:** 1 Department of Horticultural Sciences, The Islamia University of Bahawalpur, Bahawalpur, 63100 Pakistan; 2 Department of Agronomy, The Islamia University of Bahawalpur, Bahawalpur, 63100 Pakistan; 3 Department of Horticulture, Bahauddin Zakariya University, Multan, 60000 Pakistan; 4 Centre for Agriculture and Bioscience International, Rawalpindi, 46300 Pakistan; 5 Agri Development, Fauji Fresh N Freeze Ltd, Gulberg II, Lahore, 48000 Pakistan; 6 Institute of Crop Science and Resource Conservation (INRES), Crop Science, University of Bonn, 53115 Bonn, Germany; 7 Department of Seed Science and Technology, Institute of Plant Breeding and Biotechnology (IPBB), MNS-University of Agriculture, Multan, Pakistan; 8 Department of Botany and Microbiology, College of Science, King Saud University, 11451 Riyadh, Saudi Arabia

**Keywords:** *Capsicum annum* L., DPPH scavenging activities, ELE, Environment friendly, HWT, Postharvest management, Safe food additive, Storage life extension

## Abstract

**Background:**

Green chili is the predominant vegetable in tropical and subtropical regions with high economic value. However, after harvest, it exhibits vigorous metabolic activities due to the high moisture level, leading to a reduction in bioactive compounds and hence reduced shelf life and nutritional quality. Low temperature storage results in the onset of chilling injury symptoms. Therefore, developing techniques to increase the shelf life of green chilies and safeguard their nutritional value has become a serious concern for researchers. In this regard, an experiment was conducted to evaluate the impact of the alone or combined application of hot water treatment (HWT) (45 °C for 15 min) and eucalyptus leaf extract (ELE) (30%) on 'Golden Hot' chilies in comparison to the control. After treatment, chilies were stored at 20 ± 1.5 °C for 20 days.

**Results:**

HWT + ELE-treated chilies had a significant reduction in fruit weight loss (14.6%), fungal decay index (35%), red chili percentage (41.2%), soluble solid content (42.9%), ripening index (48.9%), and reactive oxygen species production like H_2_O_2_ (55.1%) and O^−2^ (46.5%) during shelf in comparison to control, followed by the alone application of HWT and ELE. Furthermore, the combined use of HWT and ELE effectively improved the antioxidative properties of stored chilies including DPPH radical scavenging activities (54.6%), ascorbic acid content (28.4%), phenolic content (31.8%), as well as the enzyme activities of POD (103%), CAT (128%), SOD (26.5%), and APX (43.8%) in comparison to the control. Additionally, the green chilies underwent HWT + ELE treatment also exhibited higher chlorophyll levels (100%) and general appearance (79.6%) with reduced anthocyanin content (40.8%) and wrinkling (43%), leading to a higher marketable fruit (41.3%) than the control.

**Conclusion:**

The pre-storage application of HWT and ELE could be used as an antimicrobial, non-chemical, non-toxic, and eco-friendly treatment for preserving the postharvest quality of green chilies at ambient temperature (20 ± 1.5 °C).

**Supplementary Information:**

The online version contains supplementary material available at 10.1186/s12870-023-04588-y.

## Background

Green chili is the most widely grown and commercially significant vegetable in tropical and subtropical regions of the world [1, 2]. Pakistan is among the five major chili-producing countries and supplies around 7.2% of the international market [3, 4]. It is a rich source of magnesium, potassium, iron, and ascorbic acid [5]. The quality maintenance of chilies is a challenge in the entire supply chain. Chilies lose a significant amount of moisture, wrinkle quickly, and lose their green color soon after the harvest. Such unfavorable changes to the quality of chilies are undesirable from the perspective of the customers [6]. In general, green chili varieties are categorized as "local" or "desi", and "hybrid" types [7]. The shelf life of fresh produce such as green chilies can be significantly increased by preserving them at low temperatures to slow down the ongoing metabolic processes [8]. But wholesale markets in the country use ambient temperatures for produce handling [6]. Moreover, green chilies are prone to chilling injury when kept below 7 °C, as green chilies are chilling-sensitive commodities [9]. The shelf life of green chilies is approximately three weeks when kept at 8–13 °C [10].

Numerous other post-harvest techniques include controlled atmosphere storage, modified atmosphere packaging, and the use of synthetic preservatives [11]. But these techniques are either expensive or their use is limited due to their potential health hazards [12]. Similarly, the use of chemicals, *i.e.,* hydrogen sulfide, sulfur dioxide, hydrogen peroxide, and calcium chloride, for shelf-life enhancement is expensive and injurious to human health [13, 14]. So, to prolong the postharvest life and retain the quality of perishable commodities, customers have been increasingly interested in treatments with antimicrobial qualities that are both affordable and effective [15].

Among various antimicrobial treatments, hot water treatment (HWT) is considered a more efficient heat transfer medium to slowing down senescence and hindering disease development [16, 17]. Heat treatments from 45 to 55 °C reduce spore germination and germ tube elongation. HWT significantly affects tissue metabolism and maintains the quality of freshly harvested commodities. HWT has been reported to effectively postpone the ripening process, improve storability, and lower the disease occurrence in peach [18], papaya [19], mango [20, 21], banana [22], sweet pepper [23, 24], and tomato [25].

Plant-based extracts have recently gained attention due to their minimal human health risks, reduced environmental impact, and affordability, making them promising alternatives to the conventional use of toxic chemicals [26]. These plant-based extracts, *i.e.,* chitosan [27, 28], propolis [29], and essential oils [30], have been shown to be effective in lowering postharvest decay and extending the storage quality of fruits and vegetables in earlier studies. These sustainable plant-based postharvest treatments can delay textural changes by decreasing rates of respiration, moisture migration, and volatile compounds loss [10]. Moreover, these extracts also serve as an effective barrier to fats and oils, demonstrating a highly selective gas permeability ratio of CO_2_/O_2_ [31].

Since eucalyptus leaf extract (ELE) has been declared safe for use in food, it is believed to prolong the shelf life of fresh produce after harvest [32]. The leaf extract of *Eucalyptus camaldulensis* var. obtusa comprises a variety of compounds, including eucalyptol/1,8-cineole (33.0%), spathulenol (21.2%), p-cymene (10.5%), γ-terpinene (6.5%), crypton (5.4%), phellandral (3.0%), thymol (2.7%), terpinen-4-ol (2.5%), cuminaldehyde (1.9%), and aromadendrene (1.1%) [33, 34]. Before employing ELE, it is essential to ascertain its antimicrobial effectiveness, composition, quantity, characteristics and suitable application method. Plant-based extracts comprising ELE or its active ingredient, *i.e.,* eucalyptol, can modify physical and biochemical quality aspects such as improving the appearance, decreasing moisture migration, sustaining firmness, limiting respiration rate, retarding cellular oxidation, preserving sensory characteristics (taste, aroma, flavor), and hindering microbial growth in several fruits, including grapes [14], passion fruit [35], nectarines [36], pears [37], mangoes [38, 39], and apples [40]. HWT and ELE are well-studied for preserving fruits and vegetables. However, their combined use to extend the storage life of green chilies while conserving quality lacks research. So, the goal of this study was to determine the influence of alone or combined use of HWT and ELE on freshly harvested green chilies to extend postharvest life by delaying senescence and maintaining bioactive compounds in the shelf (20 ± 1.5 °C).

## Results and discussion

### Fruit weight loss (%), red chilies weight (%), and fungal decay index (score)

**Fig. 1:**
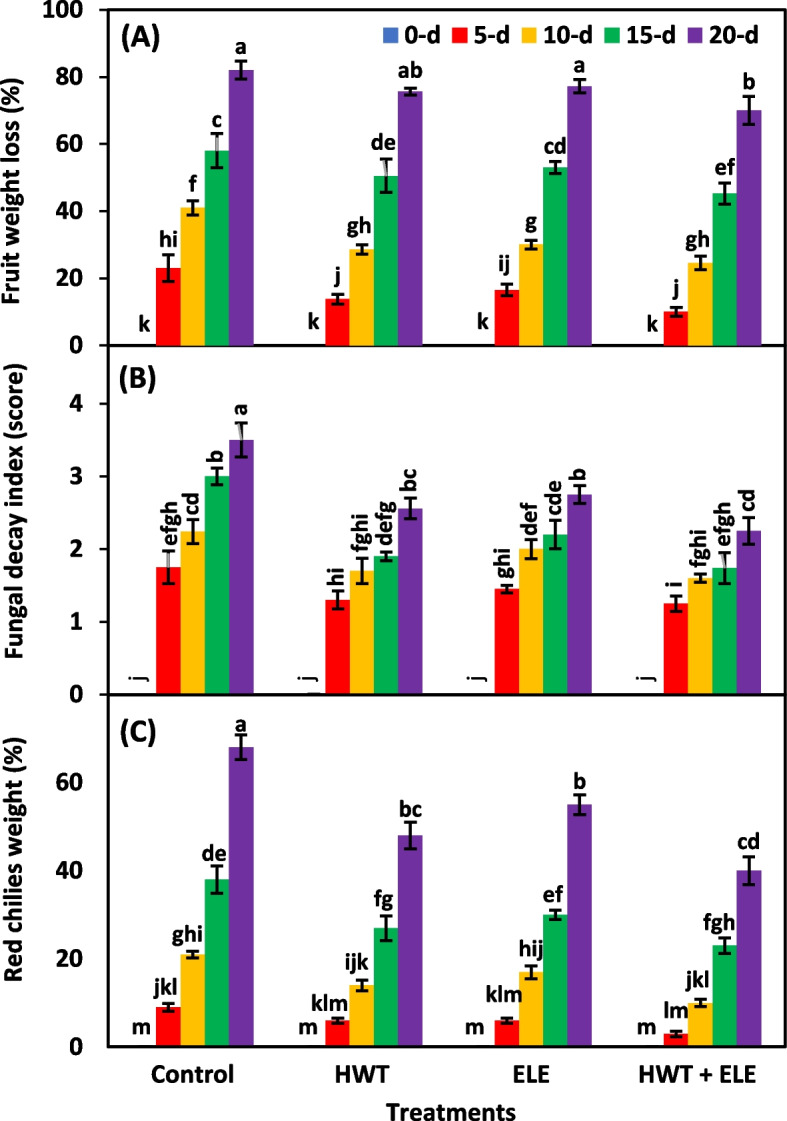
Fruit weight loss (**A**), fungal decay index (**B**), and red chilies weight (**C**) in freshly harvested and stored chilies (20 ± 1.5 °C) after application of hot water treatment (HWT) alone or in combination with eucalyptus leaf extract (ELE). The bars indicate the standard error ( ±) of the mean (*n* = 4). Lettering denotes statistical variations at the *P* ≤ *0.05*, estimated using a two-way analysis of variance (*treatment* × *storage period*)

The fruit weight loss in fresh chilies is commonly caused by the rate of respiration and moisture migration between the fruit tissues and the air around them. In the current study, the individual effects of treatments and storage periods were highly significant (*P* ≤ *0.01*) for fruit weight loss. However, their interactive effect did not significantly (*P* ≥ *0.05*) affect the weight loss of chilies (Table 1). The weight loss was significantly higher in untreated control chilies, followed by ELE and HWT. The lowest weight loss was noted in the chilies underwent HWT + ELE treatment (Fig. 1A). Generally, the weight loss of chilies significantly rose with increased storage time, regardless of treatments (Fig. 1A). On the 20^th^ day of storage, the weight loss of HWT + ELE-treated chilies was 12% lesser than that of control chilies (Fig. 1A). Green chili is one of the most highly perishable commodities due to its significant moisture loss, resulting in poor customers’ acceptance [41]. The current study has shown that green chilies treated with HWT + ELE had a much longer shelf life than those left untreated, most likely due to a delay in senescence or contamination. The results are in agreement with those of Kahramanoğlu et al. [42], who found reduced weight loss in mandarins underwent both HWT and ELE application. Our results also confirm the findings of Hazbavi et al. [41] who reported the decrease in weight loss of HWT dates stored for 6 months at room temperature. Similar findings were obtained from the combined application of hot water, salicylic acid, and calcium dipping on strawberry fruits stored for seven days at 2 °C, and lesser fruit weight loss was observed compared to the control [43]. There are a lot more studies for the evidence; for example, the shelf life of passion fruit juice was enhanced by 42.2% due to the inhibition of microbial development when the ELE applied concentration was increased from 0 to 40% [14]. In the same way, grapes treated with rosemary essential oil had a longer shelf life and less weight loss than control grapes [14, 44]. The weight loss of apples treated with 1000 µL L^−1^ of essential oil of rosemary, cinnamon, citronella, and clove was significantly reduced after 30 days of storage [37]. Similarly, the pears treated with 100 and 300 µL L^−1^ of eucalyptus and rosemary essential oils had much less fruit weight loss and longer shelf life [37]. Likewise, two nectarine cultivars exposed to ALV alone or in conjunction with thymol displayed decreased weight loss and consequently increased shelf life [36]. Mangoes dipped in aqueous plant extracts were also found to have a significant decrease in fruit weight loss and an extension of their shelf life [20, 21, 38, 39].

**Table 1:** Analysis of variance for factors (Treatment, storage period and their interaction) for fruit weight loss (WL), fungal decay index (FDI), red chilies weight (RCW), H_2_O_2_, O^−2^, ascorbic acid content (AAC), DPPH radical scavenging activities (DPPH-RSA), and total phenolic content (TPC) in chilies underwent individual hot water treatment (HWT) and eucalyptus leaf extract (ELE) or their combination (HWT + ELE) and stored for 5, 10, 15 and 20 days

Source of variance	WL	FDI	RCW	H_2_O_2_	O^−2^	AAC	DPPH-RSA	TPC
	**Percentage of total variance**							
Treatment (T)	25.01^**^	24.09^**^	28.82^**^	25.58^**^	31.40^**^	21.39^**^	24.45^**^	26.99^**^
Storage period (SP)	71.92^**^	65.05^**^	66.07^**^	62.48^**^	60.84^**^	65.94^**^	60.29^**^	68.39^**^
T × SP	0.61^*NS*^	6.07^*^	3.11^**^	10.43^**^	6.32^**^	6.70^**^	8.07^**^	2.81^**^
Error	2.07	4.52	1.96	1.49	1.40	5.54	6.75	1.69

^*^Significant at *P* ≤ *0.05*

^**^Significant at *P* ≤ *0.01*

^*NS*^Non-significant (*P *≥ *0.05)*

**Fig. 2:**
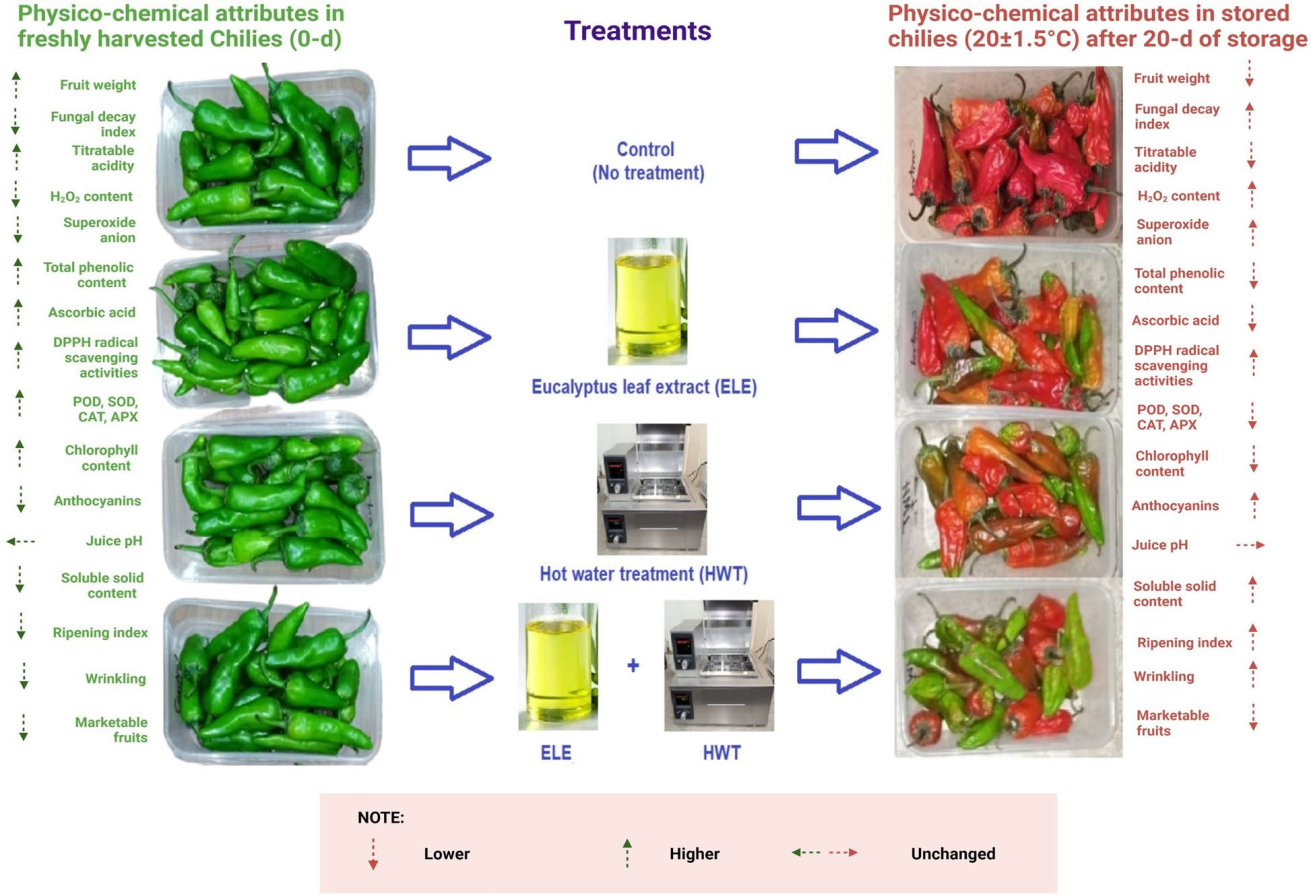
Visual difference in the appearance of freshly harvested (0-d) and stored (20 ± 1.5 °C) (20-d) chilies after individual or combined application of eucalyptus leaf extract (ELE) and hot water treatment (HWT)

To visually evaluate the fungal growth in this study, all three groups of treated and one group of non-treated green chilies were kept at an ambient temperature (20 ± 1.5 °C) (Fig. 2). The fungal decay index (FDI) was also highly significant (*P* ≤ *0.01*) for main effects and significant (*P* ≤ *0.05*) for interactive effects of treatments and storage periods (Table 1). FDI was lowest in HWT + ELE-treated chilies, followed by the individual application of HWT and ELE. Alternatively, the highest FDI was noted in the untreated control (Fig. 1B). The mycelial growth appeared on about 2.25% of the surface area of the control fruits 10 days after storage, but the same degree of fungal decay index appeared on chilies treated with HWT + ELE 20 days after storage (Fig. 1B). There has been remarkable evidence in support of our findings, i*.e.,* Rehman et al. [22] found that HWT can reduce the occurrence of microorganisms by up-regulating the activities of SOD, CAT, and APX in pepper. Similarly, Shafiee et al. [43] evaluated the combined application of hot water, salicylic acid, and calcium dipping on strawberry fruits stored at 2 °C for seven days and found lesser fruit decay than control fruits. Moreover, Al-Tayyar et al. [45] revealed that plant-based extracts have distinctive antioxidant and antibacterial properties, making them valuable additives in the food industry. In another study, Hasan et al. [6] noted that 50% of aloe vera-coated green chilies showed 13.6% lesser disease occurrence than control after the 28^th^ day when stored at 10 °C. The results of our study are also consistent with the findings of Manzi et al. [35], who found that using ELE reduced microbial loads in passion fruit. Likewise, Tyagi et al. [46] observed that applying ELE at a concentration range of 2.25–4.5 mgL − ^1^ to apple fruits reduced fungal and bacterial colony formation by 25–40%. Furthermore, Amal et al. [47] also found that using a soy protein formulation including thymol reduced microbial loads on strawberry fruits. According to Ponce et al. [48], the least bactericidal concentration of ELE application is 0.093–1.5 ml/100 (v/v), whereas the minimum inhibitory concentration is 0.049–0.13 ml/100 (v/v). Both of these doses resulted in a 99% and 90% decrease in the bacterial colony. The development of fruits' resistance to fungal infection may be attributed to the increased production of certain protective enzymes like peroxidase and chitinase due to the combined application of HWT and plant-based extracts [43].

The green chilies undergo a process of chlorophyll degradation during the later stages of maturity with the production of red pigments. The change in color from green to red is a major limitation for the longevity of its post-harvest life. The rapid deterioration of the green pigment in climacteric crops like green chilies [49] after harvest is attributed to a significant increase in both respiration rate and ethylene production [50]. In the present study, the percentage of red chilies was highly significant (*P* ≤ *0.01*) under the influence of treatments, storage periods, and their interaction (Table 1). The non-treated fruits exhibited a notable rise in the proportion of red chilies, followed by HWT and ELE. The lowest proportion of red chilies was recorded in HWT + ELE-treated group (Fig. 1C). On the 20^th^ day, the combination of HWT and ELE showed a significant decrease in red chilies weight, with a 1.7 times reduction compared to the control chilies (Fig. 1C). Our results are in accordance with previous studies that found the retention of green color in papaya [19], strawberry [31, 34, 43, 47], and grapes [14, 44] for a longer period of time after being stored. This might be related to the rapid respiration rate and ethylene production in control chilies, which resulted in the rapid degradation of the green color of the fruit into red.

### *Hydrogen peroxide (H*_*2*_*O*_*2*_*) (µmol kg*^*−1*^*) and superoxide anion (O*^*−2*^*) (nmol kg*^*−1*^*)*

**Fig. 3:**
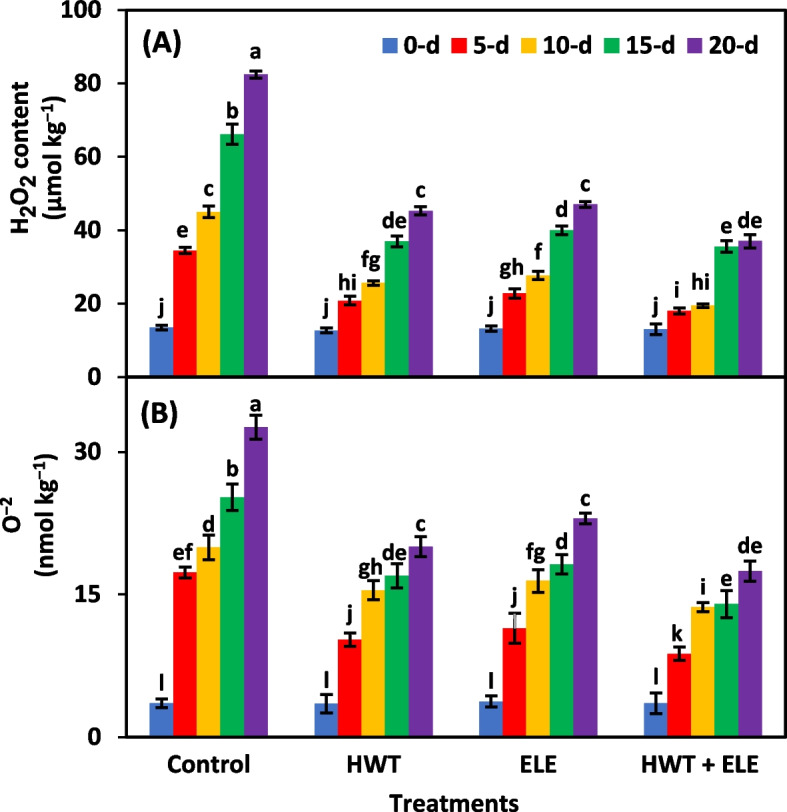
Hydrogen peroxide (H_2_O_2_) (**A**) and superoxide anion (O^−2^) (**B**) content in freshly harvested and stored chilies (20 ± 1.5 °C) after application of hot water treatment (HWT) alone or in combination with eucalyptus leaf extract (ELE). The bars indicate the standard error ( ±) of the mean (*n* = 4). Lettering denotes statistical variations at the *P* ≤ *0.05*, estimated using a two-way analysis of variance (*treatment* × *storage period*)

Treatments, storage periods, and their interactive effect were highly significant (*P* ≤ *0.01*) for H_2_O_2_ and O^−2^ (Table 1). The levels of H_2_O_2_ and O^−2^ rose with time in storage, regardless of treatments (Fig. 3A, B). However, HWT + ELE-treated chilies significantly reduced their H_2_O_2_ and O^−2^ levels than the alone application of HWT and ELE compared to the untreated control fruits (Fig. 3A, B). After 20 days of storage, HWT + ELE-treated chilies had an average of 2.23- and 1.86-times lower levels of H_2_O_2_ and O^−2^ content, respectively, than untreated control chilies (Fig. 3A, B). The findings are in accordance with those of Kahramanoğlu et al. [42], who noted reduced reactive oxygen species (ROS) production in mandarins dipped in both hot water and ELE. Recently, Hasan et al. [6] found that freshly harvested green chilies lose quality after being stored due to an accumulation of ROS. Similarly, Ali et al. [51] also noted a reduction in the quality of produce due to the buildup of ROS during storage. Consequently, post-harvest edible coatings have proven efficient in lowering ROS levels and reducing oxidative damage in fresh produce, which may allow for a longer storage and consumption window [51]. Therefore, the ELE application may lower the H_2_O_2_ and O^−2^ levels of the chilies, leading to less lipid peroxidation activity and a longer shelf life.

### Ascorbic acid content (mg kg^−1^), DPPH radical scavenging activities (%), and total phenolic content (mg kg^−1^)

**Fig. 4:**
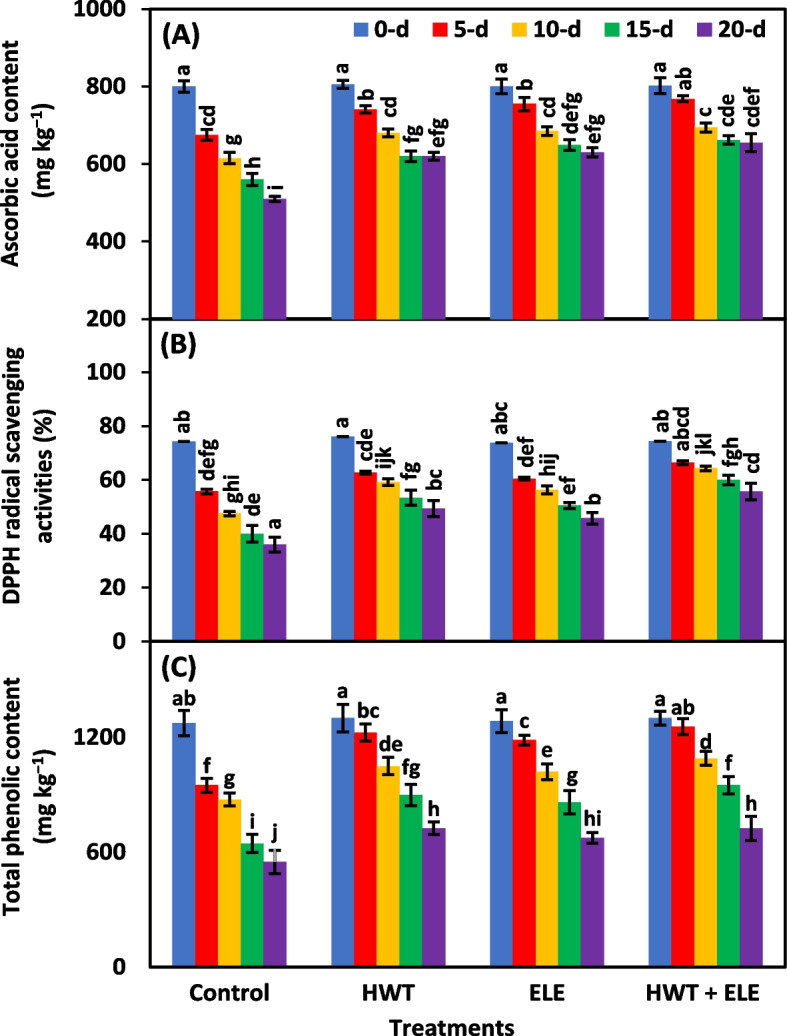
Ascorbic acid content (**A**), DPPH radical scavenging activities (**B**), and total phenolic content (**C**) in freshly harvested and stored chilies (20 ± 1.5 °C) after application of hot water treatment (HWT) alone or in combination with eucalyptus leaf extract (ELE). The bars indicate the standard error ( ±) of the mean (*n* = 4). Lettering denotes statistical variations at the *P* ≤ *0.05*, estimated using a two-way analysis of variance (*treatment* × *storage period*)

Ascorbic acid content, DPPH radical scavenging activities, and total phenolic content were highly significant (*P* ≤ *0.01*) as influenced by treatments, storage periods, and their interaction (Table 1). The levels of ascorbic acid in all the chilies, either control or treated with HWT and ELE individually or collectively, exhibited a steady decrease during storage (Fig. 4A). After 20 days of storage, ascorbic acid content dropped from 800 to 510 mg 100 g^−1^ in the control, while it dropped from 802 to 655 mg 100 g^−1^ in HWT + ELE-treated chilies (Fig. 4A). Similar results were obtained by Kahramanoğlu et al. [42], who found the highest ascorbic acid content in the mandarin fruits which received two minutes of hot water treatment with a combination of eucalyptus leaf extract. Ascorbic acid levels decrease with time after harvest due to oxidative damage in fruit tissues [52]. Application of plant-based extracts can efficiently preserve higher concentrations of ascorbic acid throughout storage by limiting oxidation processes [53]. Ebrahimi and Rastegar [54], for instance, showed that ascorbic acid content in mangoes may be retained using plant-based extracts. The application of aloe vera extract was also effective in maintaining ascorbic acid content in apricots, as reported by Nourozi and Sayyari [55], and in apples, as noted by Khan et al. [56]. Similar results with ascorbic acid content retention were reported by Rasouli et al. [57] in oranges. The positive findings were reported in both cherries [58] and litchi [51].

The radical scavenging activity of 2,2-diphenyl-1-picrylhydrazyl, decreased over time in all three groups of treated chilies and control (Fig. 4B). However, the DPPH-RSA of HWT + ELE-treated chilies was significantly higher than control chilies, followed by HWT and ELE-treated chilies (Fig. 4B). On the 20^th^ day, HWT + ELE-treated chilies had a greater DPPH-RSA (51.34%) than untreated control chilies (Fig. 4B). Higher DPPH-RSA preservation during storage has been shown to reduce oxidative stress [51]. Free radicals produced during postharvest storage may be responsible for the general decline in total antioxidants of fresh food. Exogenous ELE application has shown promising results in preserving DPPH-RSA in several other fruits and vegetables, including papayas [19], strawberries [31, 34, 43, 47], grapes [14, 44], and tomatoes [25, 59]. Increased DPPH-RSA after a longer storage duration in HWT + ELE-treated chilies can be attributed to the lowered formation of free radicals and the postponement of senescence. The decline in DPPH-RSA preservation of untreated chilies might be due to the production of free radicals due to the early onset of senescence and decay.

Total phenolic content (TPC) progressively dropped over the course of the storage period (Fig. 4C). On average, HWT + ELE application significantly lowered the decrease in TPC of chilies compared with untreated ones, followed by the alone application of HWT and ELE. After 20 days of storage, about 1.32 times higher TPC (725 mg kg^−1^) were recorded in HWT + ELE-treated chilies in contrast to the untreated control (549.8 mg kg^−1^) (Fig. 4C). Results indicated that the HWT + ELE treatment prevented oxidative damage due to increased TPC [60]. Secondary metabolites present in plant tissues, such as phenolic substances (phenolic acids, tannins, lignin, phenylpropanoids, quinones, hydroxycinnamate esters, and flavonoids), are thought to have antioxidant properties. So, the phenolic substances they produce are beneficial in order to counteract oxidative stress [61]. Our findings for increased TPC in HWT + ELE-treated chilies validated the prior findings that combined application of HWT and plant-based extracts reduced the loss of TPC in apples [37, 40, 56], strawberries [31, 34, 43, 47], tomatoes [25, 59], and nectarines [36]. It has been suggested that a greater TPC content may help prevent the spread of postharvest diseases while in storage [61]. As a result, HWT and ELE together have potential as a treatment applied after harvest to postpone senescence and lessen TPC loss in storage.

### APX, CAT, POD, and SOD enzyme activities (µmol s^−1^ kg^−1^)

**Fig. 5:**
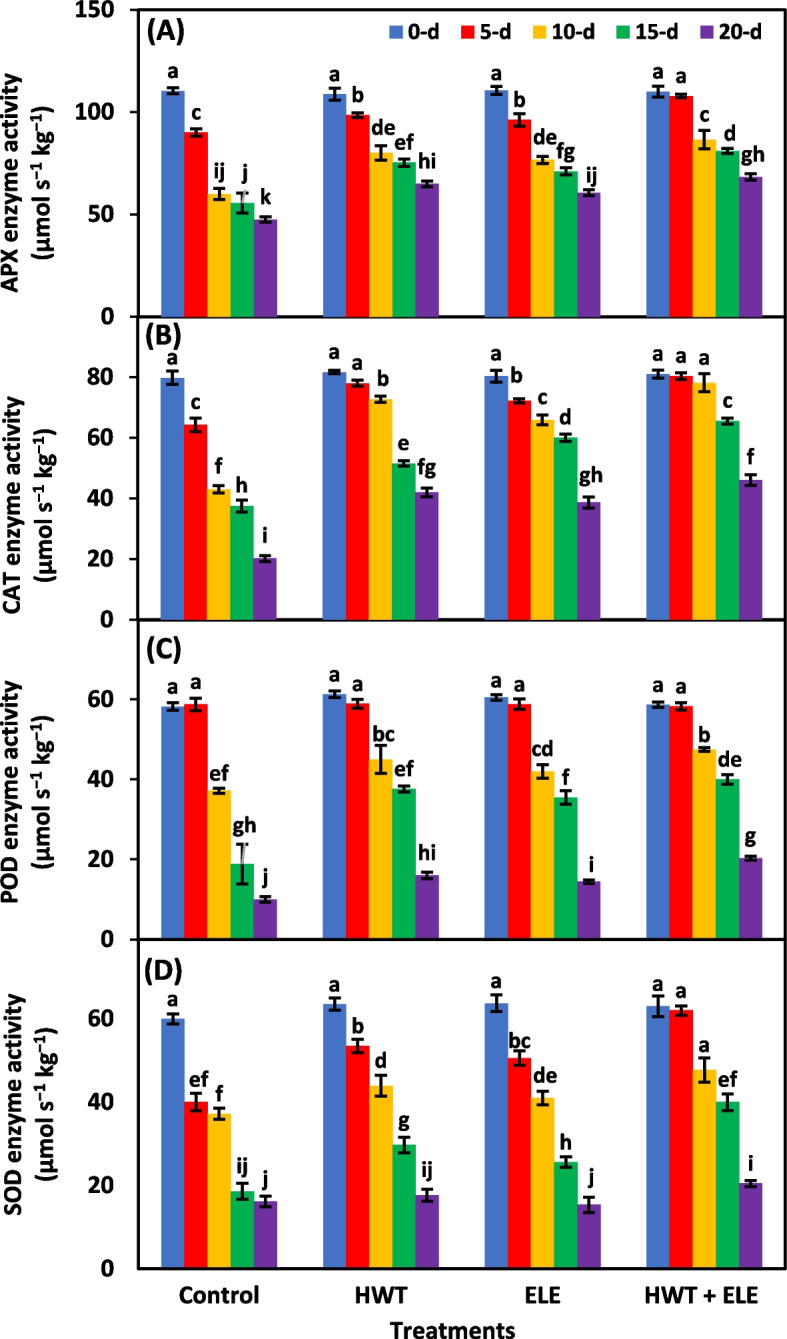
Ascorbate peroxidase (**A**), catalase (**B**), superoxide dismutase (**C**), and peroxidase (**D**), enzyme activities in freshly harvested and stored chilies (20 ± 1.5 °C) after application of hot water treatment (HWT) alone or in combination with eucalyptus leaf extract (ELE). The bars indicate the standard error ( ±) of the mean (*n* = 4). Lettering denotes statistical variations at the *P* ≤ *0.05*, estimated using a two-way analysis of variance (*treatment* × *storage period*)

The individual effects of treatments, storage periods and their interaction were highly significant (*P* ≤ *0.01*) for APX, CAT, POD, and SOD enzyme activities (Table 2). The ascorbate peroxidase (APX) and superoxide dismutase (SOD) enzyme activities exhibited a gradual decrease in chilies during the entire storage period, irrespective of the treatments applied (Fig. 5C, D). Overall, chilies treated with HWT + ELE exhibited 1.43- and 1.26-fold increases in APX (Fig. 5A) and SOD (Fig. 5D) enzyme activities, respectively, compared to the untreated control fruits after 20 days of storage. Mangena and Muyima [62] also found that SOD plays a crucial role in maintaining the quality of mushrooms, which was achieved through the elimination of free oxygen radicals and protecting the integrity of the mushrooms' membranes against oxidative stress. Catalase (CAT) activity exhibited a swift decline over the first ten days of storage in all treated and non-treated chilies (Fig. 5B). However, the CAT activity subsequently showed a slow decrease for all four groups until the final day of storage (Fig. 5B). Similar findings were reported by Haider et al. [63], who reported 1.7 times higher CAT activity in ELE-treated strawberries 15 days after storage compared to control fruits. Our study also confirms the findings of Zhao et al. [64], who found enhanced activities of ROS-scavenging enzymes in HWT peaches compared to control. The activity of peroxidase (POD) enzymes exhibited a slow decline over the first five days of storage, then a rapid decline gradually until the last day of storage (day-20) (Fig. 5C). However, chilies treated with HWT + ELE showed all-time enhanced enzyme activity compared to control, followed by HWT and ELE (Fig. 5C). After 20 days of storage, the chilies treated with HWT + ELE showed a two-fold increase in POD activity compared to the untreated control chilies (Fig. 5A). Similar findings were obtained by Badawy et al. [59], who noted that essential oils containing geraniol (0.04%) or thymol (0.02%) increased POD activities. POD is a prooxidant enzyme found in organelles that differs significantly from phenolic substances, restricts lipid peroxidation, and safeguards against DNA hydroperoxides in fruits and vegetables [65]. Therefore, the use of HWT and ELE has the potential to effectively preserve membrane integrity by maintaining the elevated activity of POD enzymes throughout storage. Overall, these results suggest that the combined use of HWT and plant-based extracts in freshly harvested produce increases oxyradical detoxification enzyme levels, *i.e.*, POD, CAT, SOD, and APX.

**Table 2:** Analysis of variance for factors (Treatment, storage period and their interaction) for enzyme activities of ascorbate peroxidase (APX), catalase (CAT), peroxidase (POD), and superoxide dismutase (SOD), and juice pH, ripening index (RI), soluble solid content (SSC), and titratable acidity (TA) in chilies underwent hot water treatment (HWT), eucalyptus leaf extract (ELE) or their combination (HWT + ELE) and stored for 5, 10, 15 and 20 days

Source of variance	APX	CAT	POD	SOD	Juice pH	RI	SSC	TA
	**Percentage of total variance**							
Treatment (T)	23.68^**^	18.74^**^	24.37^**^	20.67^**^	6.06^*NS*^	43.40^**^	43.19^**^	23.78^**^
Storage period (SP)	70.12^**^	72.21^**^	70.89^**^	74.50^**^	1.27^*NS*^	42.54^**^	41.11^**^	68.17^*^
T × SP	3.62^**^	6.92^**^	3.16^**^	3.11^**^	0.30^*NS*^	7.21^**^	6.71^**^	1.50^*NS*^
Error	2.56	1.84	1.45	1.70	83.5	4.37	4.85	4.91

^*^Significant at *P* ≤ *0.05*

^**^Significant at *P* ≤ *0.01*

^*NS*^Non-significant (*P* ≥ *0.05)*

### Juice pH, ripening index, SSC (%), and TA (%)

**Fig. 6:**
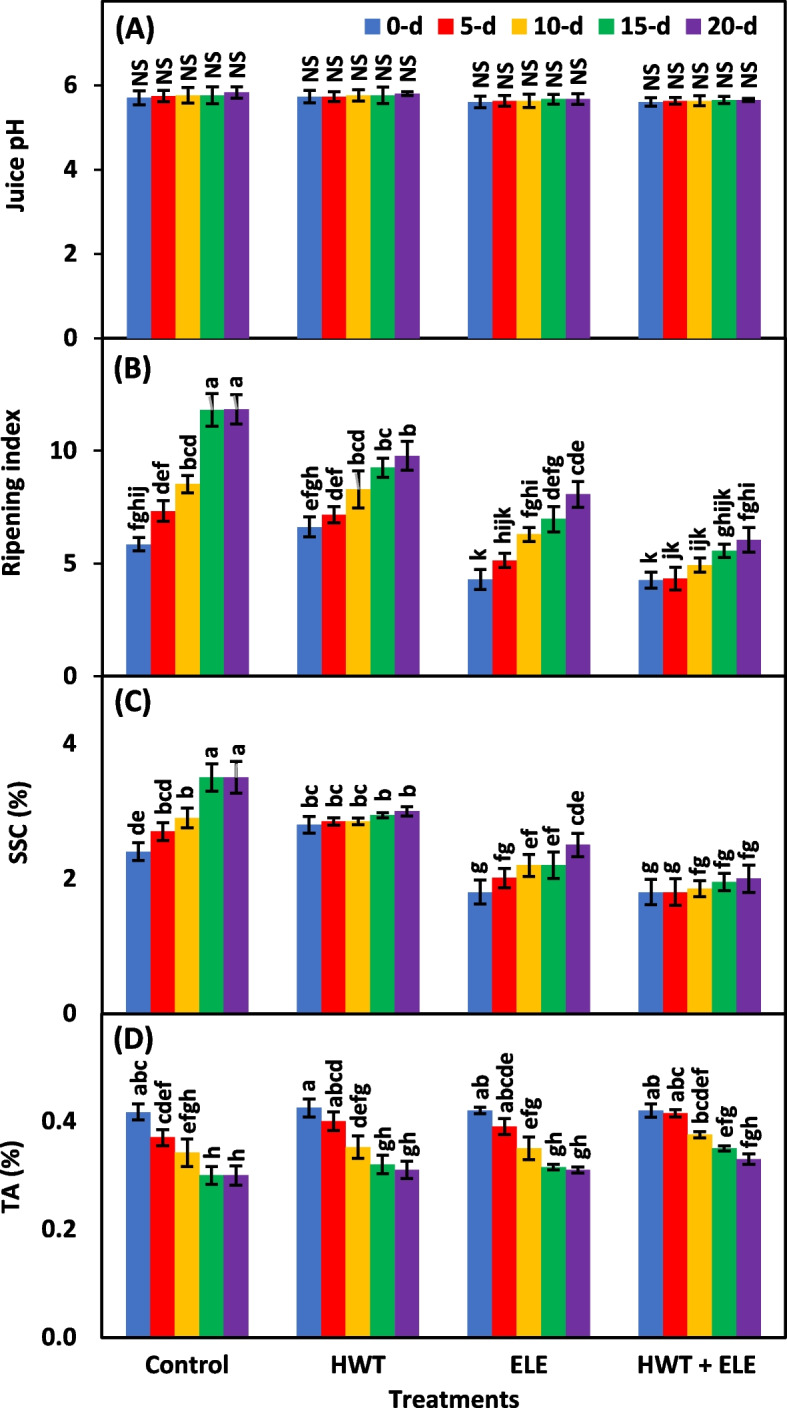
Juice pH (**A**), ripening index (**B**), soluble solid content (**C**), and titratable acidity (**D**) of freshly harvested and stored chilies (20 ± 1.5 °C) after application of hot water treatment (HWT) alone or in combination with eucalyptus leaf extract (ELE). The bars indicate the standard error ( ±) of the mean (*n* = 4). Lettering denotes statistical variations at the *P* ≤ *0.05*, estimated using a two-way analysis of variance (*treatment* × *storage period*)

The main and interactive effects of treatments and storage periods were found to be non-significant (*P* ≥ *0.05*) for the juice pH of chilies (Table 2). Although the juice pH was lower in the HWT + ELE-treated chilies compared to the control (Fig. 6A), which confirms the findings of Haider et al. [63], who also reported a lower juice pH in the ELE-treated fruits compared to the control fruits. The ripening index, representing the ratio of soluble solid content (SSC) and titratable acidity (TA), was highly significant (*P* ≤ *0.01*) under the main and interactive effects of treatments and storage periods (Table 2). The ripening index elevated in this study over the course of storage; however, by the 20^th^ day, HWT + ELE-treated fruits were at a lower level (6.05) than the control (11.85) (Fig. 6B). In recent studies, sole or combined application of HWT and ELE decreased the sugar-to-acid ratio in strawberries [31, 34, 43, 47, 63], grapes [14, 44], pears [37], apples [37, 40], and mangoes [20, 21, 38, 39, 54, 61] during storage. SSC was also highly significant (*P* ≤ *0.01*) under the effects of treatments, storage periods, and their interaction (Table 2). SSC steadily increased during the entire storage period, irrespective of treatments (Fig. 6C). The average increase in SSC of chilies was lowest in the HWT + ELE group, followed by ELE, HWT, and then control (Fig. 6C). Previously, similar findings were obtained as increased SSC in apples [37, 40, 56], strawberries [31, 34, 43, 47, 63], tomatoes [25, 59], and litchi [51] in response to applied plant-based extracts during storage. In this study, TA was highly significant (*P* ≤ *0.01*) under treatments effect and significant (*P* ≤ *0.05*) under storage periods effect, while was non-significant (*P* ≥ *0.05*) under their interactive effect (Table 2). TA decreased with the progression of the storage period, but the decrease was slightly lower in HWT + ELE-treated chilies (0.46%) compared to the control (0.35%) after 20 days of storage (Fig. 6D). The findings are in agreement with those of Haider et al. [63], who noticed a significantly lower reduction in ELE-treated strawberries compared to the control during 15 days of storage. Alternatively, the findings of Vieira et al. [40] contradict our results, who found non-significant variations in the TA of apples after the application of cinnamon, clove, citronella, and rosemary. Tzortzakis [66] likewise observed non-significant differences in the TA of tomatoes and strawberries exposed to eucalyptus and cinnamon fumes for 10 days.

### Anthocyanin and chlorophyll content (mg Kg^−1^ FW)

**Fig. 7:**
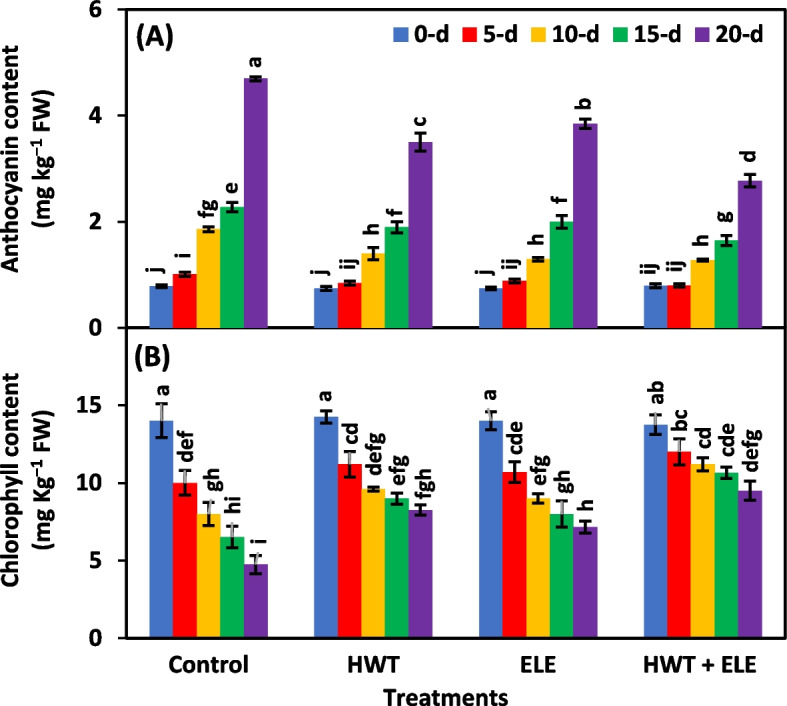
Anthocyanin (**A**) and chlorophyll (**B**) content of freshly harvested and stored chilies (20 ± 1.5 °C) after application of hot water treatment (HWT) alone or in combination with eucalyptus leaf extract (ELE). The bars indicate the standard error ( ±) of the mean (*n* = 4). Lettering denotes statistical variations at the *P* ≤ *0.05*, estimated using a two-way analysis of variance (*treatment* × *storage period*)

The main effects of treatments and storage periods were highly significant (*P* ≤ *0.01*) for anthocyanin and chlorophyll content in chilies (Table 3). However, the interactive effect was significant (*P* ≤ *0.05*) for only anthocyanin and non-significant (*P* ≥ *0.05*) for chlorophyll content (Table 3). The anthocyanin content was linearly increased in all treated and non-treated chilies as the storage time advanced; nevertheless, the content rose at a faster rate in the untreated group than in the HWT + ELE, HWT, or ELE-treated group (Fig. 7A). The changes in chlorophyll and anthocyanin content were consistent with earlier studies carried out on the postharvest preservation effects of plant-based extracts on strawberries [31, 34, 47, 63], plums [67], peaches [18, 64], passion fruits [35], and nectarines [36]. Thus, HWT and ELE application together can preserve the levels of chlorophyll and delay the accumulation of anthocyanins by postponing fruit senescence, modulating oxidative stress, and preventing the breakdown of coloring pigments. The HWT + ELE-treated chilies substantially retained chlorophyll content compared to untreated control chilies throughout a 20-day storage period (Fig. 7B). However, the chlorophyll content in control chilies declined at a faster rate than the HWT + ELE-treated chilies (Fig. 7B). On the 20^th^ day of storage, the chlorophyll content was twice as high (9.50 mg Kg^−1^ FW) as untreated control chilies (4.75 mg Kg^−1^ FW) (Fig. 7B). The presence of chlorophyll gives fresh chilies their characteristic green color, which is an indication of their peak eating quality [6]. The slow deterioration of chlorophyll in HWT + ELE-treated chilies is directly related to the slow respiration and oxidation of specific enzyme processes, causing the green color to sustain with increasing storage time.

**Table 3:** Analysis of variance for factors (Treatment, storage period and their interaction) for anthocyanin content, chlorophyll content, general appearance, marketable fruits, and wrinkling in chilies underwent hot water treatment (HWT), eucalyptus leaf extract (ELE) or their combination (HWT + ELE) and stored for 5, 10, 15 and 20 days

Source of variance	Anthocyanin content	Chlorophyll content	General appearance	Marketable fruits	Wrinkling
	**Percentage of total variance**				
Treatment (T)	24.55^*^	22.87^*^	20.01^*^	14.54^*^	12.94^*^
Storage period (SP)	69.23^*^	65.93^*^	70.38^*^	72.86^*^	68.62^*^
T × SP	4.74^*^	5.40^*NS*^	5.03^*^	7.06^*^	12.58^*^
Error	1.37	4.44	3.97	5.45	5.29

^*^Significant at *P* ≤ *0.01*

^*NS*^Non-significant (*P* ≥ *0.05)*

### General appearance (score), marketable fruits (%) and wrinkling (score)

**Fig. 8:**
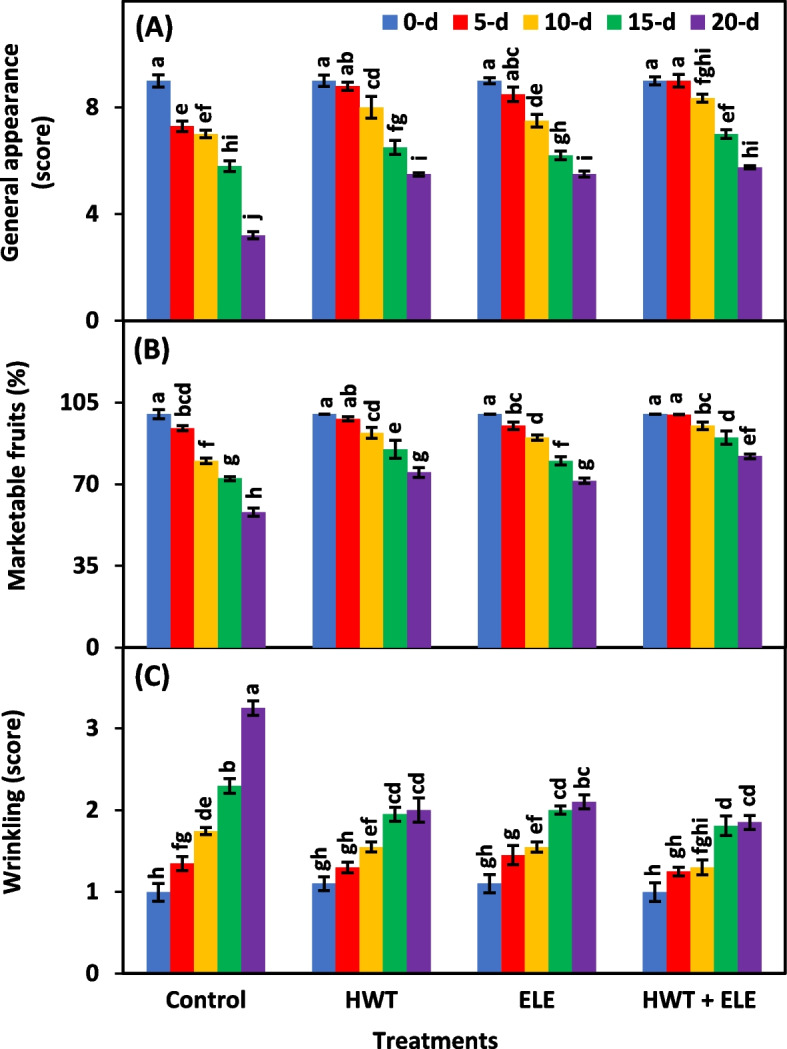
General appearance (**A**), marketable fruits percentage (**B**), and Wrinkling (**C**) of freshly harvested and stored chilies (20 ± 1.5 °C) after application of hot water treatment (HWT) alone or in combination with eucalyptus leaf extract (ELE). The bars indicate the standard error ( ±) of the mean (*n* = 4). Lettering denotes statistical variations at the *P* ≤ *0.05*, estimated using a two-way analysis of variance (*treatment* × *storage period*)

Treatments, storage periods, and their interactive effect were highly significant (*P* ≤ 0.01) for general appearance, marketable fruits percentage, and wrinkling (Table 3). The general appearance of HWT + ELE treated chilies was 1.8 times better than the untreated control on the last day of storage (Fig. 8A). Similarly, the HWT + ELE-treated chilies exhibited lower wrinkling and better visual appeal, which led to a higher proportion of marketable fruits compared to the untreated control group, followed by the individual application of HWT and ELE (Fig. 8A–C). The HWT + ELE-treated group of chilies had a higher percentage of marketable fruit (82.2%) than the control group (57.8%) at the last removal (20^th^ day) from storage (Fig. 8B). The visual quality of chillies is adversely impacted by the increased wrinkling occurring during storage [68]. The wrinkling at the 20^th^ day of storage was 43% lower in HWT + ELE-treated chilies than the untreated control (Fig. 8C). The application of plant-based extracts has been found to maintain the general appearance and reduce wrinkling in various fruits and vegetables. This effect has been observed in strawberries [31, 34, 43, 47, 63, 69], papaya [70], peaches [71], oranges [57], apples [69], sapodilla [72], nectarines [73], and tomatoes [74] throughout the entire storage period.

## Conclusion

The combined application of hot water treatment (HWT) and eucalyptus leaf extract (ELE) on green chilies before storage significantly prolonged their shelf life by decreasing the fungal decay index, percentage of red chilies, soluble solid content, ripening index, and ROS production. Moreover, the combined use of HWT and ELE preserved chilies' antioxidative properties (DPPH scavenging activities, ascorbic acid content, phenolic content, POD, CAT, SOD, and APX enzyme activities) during storage. The green chilies treated with HWT + ELE maintained their chlorophyll levels and general appearance with reduced anthocyanin content and wrinkling, resulting in a greater proportion of marketable fruits. So, HWT (45 °C for 15 min) and ELE [30% (v/v)] together could be used as a sustainable non-chemical, non-toxic, and eco-friendly treatment to preserve the postharvest quality of green chilies during storage at 20 ± 1.5 °C. The implementation of this approach on a commercial scale could help reduce food waste and promote the use of eco-friendly preservation methods in the food industry. Further research on the gene-expression of physicochemical traits will elucidate the mechanism of HWT and ELE impact on chilies postharvest.

## Methodology

### Source of plant material

Freshly harvested green chilies (*Capsicum annum* L.) of cv. “Golden Hot” were collected from the Horticulture Experimental Area (29°22′17.4″ N 71°45′53.6″ E), Department of Horticultural Sciences (DoHS), The Islamia University of Bahawalpur (IUB), Pakistan. The chilies were picked when they reached commercial maturity (100% green). After harvesting, the chilies were immediately shifted to the General Laboratory (GL), DoHS, IUB within 20 min. The defect-free chilies were rinsed with clean water after harvesting. The surface of selected chilies was dried at 25 °C after three minutes of washing with 0.1% sodium hypochlorite to reduce microbiological contamination. There were three treatment groups, *i.e.,* hot water treatment (HWT), eucalyptus leaf extract (ELE), and HWT + ELE, along with a single control group. A total of 800 fruits were selected for this study, with 50 fruits in each box, as each one of the four replications contained one plastic box comprising 200 fruits per group.

### Hot water treatment (HWT)

For HWT, chilies were dipped in 45 °C hot water for 15 min and kept cool to 20 °C, as reported by Endo et al. [23]. After HWT, chilies were dried in the air and then kept at 20 ± 1.5 °C with 85–90% RH for 20 days.

### Eucalyptus leaf extract (ELE) solution preparation

The young leaves of 15 years old tree of *Eucalyptus camaldulensis* var. obtusa (of the same color, size, freshness, and maturity levels) were obtained from the Forestry Research Area (29°37′17.4′′ N 71°76′53.6′′ E), IUB, and then transported to GL, DoHS, IUB. After that, the selected leaves were washed off with running water to eliminate insects, phytoplankton, and dirt. Then, they were sliced into small pieces using scissor. Following a three-minute soaking in a 0.1% sodium hypochlorite solution, the sliced parts were allowed to entirely air dry at room temperature. After adding them into water, they were preheated to 55 °C using hot water bath (WNB-45, Memmert, Schwabach, Germany) and the temperature was raised to 90 °C. It was held at that temperature for 10 min before being cooled to 55 °C. Subsequently, the sliced pieces of leaves were crushed and homogenized by a blender (Kenwood, KW-3500, Havant, UK). The collected juice was strained using a muslin cloth in order to remove the spongy material. To maintain the pH level of the juice below 3.5, citric acid was used at a concentration of 1% (v/w) [35]. After that, it underwent a 45-min pasteurization process at 70 °C [36]. The ELE solution was allowed to cool to ambient temperature before being diluted with distilled water in a 1:1 (v/v) for later use. The chilies were dipped in ELE solution (30%) for a duration of five minutes. The 30% concentration of ELE was selected on the basis of a preliminary assessment with 0, 15, 30, 45, and 60% solutions. The chilies that underwent ELE treatment were finally air-dried and kept at 20 ± 1.5 °C for 20 days with a RH of 85–90%.

### Fruit weight loss (%), fungal decay index (score), and red chilies weight (%)

In order to determine the fungal decay index (FDI), the fruits were visually inspected. The FDI was then calculated, and the fruits were given a rating as a percentage of the rotted area of the fruit, where 1 = no fruit decay; 2 = 5% of the fruit area under decay; 3 = 6–20% of the fruit area under decay; 4 = 21–50% of the fruit area under decay; and 5 =  > 50% of the surface area under decay. The red chilies were weighed using a digital weighing balance (DM-01, ScaleTech, Beijing, China) at the end of each removal, *i.e.,* 5, 10, 15, and 20 days of storage. The below equation was used to compute the weight of red chilies, and the results were expressed as a percentage. For assessment of fruit weight loss, the weight of fruits was recorded on the first and last day of each removal. The weight loss of all treated and non-treated control chilies was estimated by calculating the difference between their final and initial weights, as previously explained by Ali et al. [60].

$$\mathrm{Red\,chili\,percentage}=\frac{\mathrm{Red\,chilies\,weight\,}}{\mathrm{Total\,chilies\,weight}}\,\times\,100$$

### Hydrogen peroxide (H_2_O_2_) (µmol kg^−1^) and superoxide anion (O^−2^) (nmol kg^−1^)

For the determination of H_2_O_2_ content, the method outlined by Ali et al. [60] was adopted, in which 1 g of chilies were ground in 1 ml of 0.1% TCA and centrifuged for 15 min at *12,000* × *g*. After that, 0.5 ml of extracted supernatant was mixed with 10 mmol/L of phosphate buffer (pH = 7) and 1 M KI. Subsequently, the reading was taken by determining the absorbance of every sample at 390 nm and expressed as μmol/kg of fresh weight.

The amount of O^−2^ produced was estimated using a method reported by Hasan et al. [6]. A sample of 1 g of chili was homogenized with 3 ml of phosphate buffer coupled with 1% polyvinylpyrrolidone at 4 °C. After that, the samples were centrifuged at *10,000* × *g* for 15 min. They were then mixed with 10 mmol L^–1^ hydroxylamine hydrochloride and incubated at 25 °C for half an hour. The sample’s absorption was detected at 530 nm. The amount of O^−2^ was estimated by using the NO_2_ curve as a standard, and the values were presented in nmol/kg FW.

### Antioxidative enzyme assays

The chiles were ground in 2 ml of phosphate buffer (pH 7.2) using a cold mortar and pestle. The mixture was then spun in a centrifuge (Rotofix 46, Hettich, Kirchlengern, Germany) at *10,000* × *g* for five minutes at 4 °C. After collecting the supernatant, the activities of antioxidative enzymes were recorded. Peroxidase (POD) (EC 1.11.1.7), catalase (CAT) (EC 1.11.1.6), and superoxide dismutase (SOD) (EC 1.15.1.1) activities were determined by adopting the method earlier described by Ali et al. [60], and samples were read at different wavelengths, *i.e.,* 470, 240, and 560 nm, respectively. Ascorbate peroxidase (APX) (EC 1.11.1.11) was examined using the method of Nakano and Asada [75], in which the oxidation-induced absorption of ascorbic acid was recorded at 290 nm. The enzyme activities were expressed in terms of µmol kg^−1^ FW.

### Biochemical quality

For the assessment of the biochemical quality of chilies, total soluble solid content was evaluated from extracted juice using a digital refractometer (RX-5000, Atago, Tokyo, Japan) and presented as a percentage. Titratable acidity was quantified by titration against a 0.4% sodium hydroxide solution with phenolphthalein as an indicator, as before established by Hassan et al. [31]. The sugar-acid ratio, commonly known as the ripening index, was computed by dividing SSC values by TA. The pH of the chili juice was determined using a digital pH meter (HI98130, Hanna, Nușfalău, Romania) [76]. The quantification of total phenolic content (TPC) in chilies was carried out using the Folin-Ciocalteu reagent after taking absorbance at a wavelength of 765 nm [77]. Standard curve of gallic acid was plotted and concentration of TPC was expressed as mg kg^−1^. This quantification was achieved by measuring the absorbance at 765 nm. The ascorbic acid content in chilies was determined using the protocol of Ali et al. [53]. In this method, a 10 mL aliquot of chili juice was taken in a flask and diluted to a total volume of 100 mL using a 0.4% oxalic acid solution. A 5 mL aliquot was then collected and subjected to titration with 2,6-dichloroindophenol. Finally, the ascorbic acid content was calculated and expressed as mg 100 g^−1^ FW. The scavenging activity against 2,2-diphenyl-1-picrylhydrazyl (DPPH) radicals was measured using the method published by Ali et al. [56], and the results were expressed as a percentage of inhibition. The chlorophyll content of chilies were determined by adopting the method of Zhang et al. [78]. The approach of Zheng and Tian [79] was used to quantify total anthocyanin content in chilies. In this method, one gram of sample was extracted with HCl-methanol solution (15:85) in a shaking water bath at 25 °C for 6 h. The sample was then centrifuged at *4000* × *g* for 20 min at 4 °C in a temperature-controlled centrifuge. A UV–Vis spectrophotometer (2326 K, Hermle Labortechnik GmbH, Wehingen, Germany) was used to detect the absorbance at 650, 620, and 530 nm. The following formula was used to calculate total anthocyanins (mg Kg^−1^ FW):

$$\mathrm{Anthocyanins\,}=\,(\mathrm{A}530\mathrm{\,nm\,}-\mathrm{\,A}620\mathrm{\,nm})\,-\,0.1\,(\mathrm{A}650\mathrm{\, nm}-\mathrm{\,A}620\mathrm{\,nm})$$

### Wrinkling (score), general appearance (score), and marketable fruits (%)

Wrinkling was evaluated using the visual symptoms of dehydration outlined by Hameed et al. [65]. Wrinkling was scored on a scale of 1 to 4, with 1 indicating no sign, 2 indicating some, 3 indicating moderate, and 4 indicating severe signs. The general appearance was assessed by analyzing the samples. The samples’ placement and labelling were at random on individual plastic boxes. The appearance of chilies was rated using a scale from 1 to 9 [80]: 1 for poor quality, 3 for fair (limit of usability), 5 for good (limit of marketability), 7 for very good, and 9 for excellent and fresh-looking with intermediate numbers assigned where appropriate. To calculate the marketable fruit percentage, healthy, disease-free, and decay-free fruits were visually observed, weighed for each group, and expressed as a percentage [5].

### Statistical analysis

All data processing was carried out on Microsoft Excel 2016. The experiment was arranged according to a completely randomized design with two-factor factorial settings. The analysis of variance (ANOVA, general linear model) was implemented to find the significant difference, while the least significant difference test was used to evaluate the magnitude of the difference between treatment means at *p* ≤ *0.05* in statistical software, Statistix 9® (Analytical Software, Tallahassee, CA, USA). A total of four replicates (*n* = 4) were used to conduct a statistical analysis of all parameters.

### Supplementary Information

**Additional file 1: Fig. S1.** Effect of different eucalyptus leaf extract concentrations on fruit weight loss in chilies. **Fig. S2.** Effect of different eucalyptus leaf extract concentrations on fungal decay index in chilies. **Fig. S3.** Effect of different eucalyptus leaf extract concentrations on marketable fruits in chilies. **Fig. S4.** Effect of different eucalyptus leaf extract concentrations on red chilies weight. **Table S1.** Physicochemical analysis of eucalyptus leaf extract used in this study.

## Data Availability

All the data related to this work can be sourced from the corresponding authors.

## Abbreviations

APX: Ascorbate peroxidase
CAT: Catalase
DoHS: Department of Horticultural Sciences
DPPH-RSA: 2,2-Diphenyl-1-picryl-hydrazyl-hydrate-radical scavenging activity
ELE: Eucalyptus leaf extract
FDI: Fungal decay index
GL: General Laboratory
HWT: Hot water treatment
IUB: The Islamia University of Bahawalpur
POD: Peroxidase
RH: Relative humidity
SOD: Superoxide dismutase
SSC: Soluble solid content
TA: Titratable acidity
TPC: Total phenolic content
