# Supplementary material for: Combined application of hot water treatment and eucalyptus leaf extract postpones seneṣcence in harvested green chilies by conserving their antioxidants: a sustainable approach
Source: BMC Plant Biol. 2023 Nov 18;23:576. doi: 10.1186/s12870-023-04588-y (PMC10656992; doi:10.1186/s12870-023-04588-y)
Supplement: Supplementary file 1 — Additional file 1: Fig. S1. Effect of different eucalyptus leaf extract concentrations on fruit weight loss in chilies. Fig. S2. Effect of different eucalyptus leaf extract concentrations on fungal decay index in chilies. Fig. S3. Effect of different eucalyptus leaf extract concentrations on marketable fruits in chilies. Fig. S4. Effect of different eucalyptus leaf extract concentrations on red chilies weight. Table S1. Physicochemical analysis of eucalyptus leaf extract used in this study. [file 12870_2023_4588_MOESM1_ESM.docx]

1. **Selection criteria for ELE concentration during preliminary assessment**

The optimization of ELE concentration was carried out based on physical attributes including fruit weight loss, fungal decay index, marketable fruits percentage and red chilies weight percentage.

- 1. **Fruit weight loss**

**Fig. S1.** Effect of different eucalyptus leaf extract concentrations on fruit weight loss in chilies.

- 1. **Fungal decay index**

**Fig. S2.** Effect of different eucalyptus leaf extract concentrations on fungal decay index in chilies.

- 1. **Marketable fruits**

**Fig. S3.** Effect of different eucalyptus leaf extract concentrations on marketable fruits in chilies.

- 1. **Red chilies weight**

**Fig. S4.** Effect of different eucalyptus leaf extract concentrations on red chilies weight.

1. **Physicochemical composition of experimental eucalyptus leaf extract**

**Table S1.**  [Physicochemical analysis of eucalyptus](https://biolres.biomedcentral.com/articles/10.1186/0717-6287-48-7) leaf extract used in this study.

| **Particular** | **Unit** | **Value** |
| --- | --- | --- |
| Moisture content | % | 34.0 |
| pH | - | 5.5 |
| Color | - | yellowish |
| Refractive index | - | 1.40 |
| Total phenolic content | mg GAE g^−1^ extract | 130.5 |
| DPPH scavenging activities | % | 89.4 |
| Eucalyptol | % | 29.6 |
| Total flavonoid content | mg QE g^−1^ extract | 27.8 |
| γ-terpinene | % | 5.2 |
| Hydroxycinnamic acid | mg CAE g^−1^ extract | 2.5 |
| Thymol | % | 2.0 |
| Proanthocyanidins | mg EE g^−1^ extract | 0.17 |
